# Changes in circulating exosome molecular profiles following surgery/(chemo)radiotherapy: early detection of response in head and neck cancer patients

**DOI:** 10.1038/s41416-021-01567-8

**Published:** 2021-10-12

**Authors:** M.-N. Theodoraki, S. Laban, E. K. Jackson, R. Lotfi, P. J. Schuler, C. Brunner, T. K. Hoffmann, T. L. Whiteside, L. Hofmann

**Affiliations:** 1 Department of Otorhinolaryngology, Head and Neck Surgery, Ulm University Medical Center, 89075 Ulm, Germany; 2 Department of Pharmacology and Chemical Biology, University of Pittsburgh School of Medicine, Pittsburgh, PA USA; 3 Institute for Clinical Transfusion Medicine and Immunogenetics Ulm, German Red Cross Blood Services Baden-Württemberg-Hessen, 89081 Ulm, Germany; 4 Institute for Transfusion Medicine, University Hospital Ulm, 89081 Ulm, Germany; 5 Department of Pathology, University of Pittsburgh School of Medicine, Pittsburgh, PA USA; 6 UPMC Hillman Cancer Center, Pittsburgh, PA USA; 7 Department of Medicine, University of Pittsburgh School of Medicine, Pittsburgh, PA USA; 8 Department of Immunology, University of Pittsburgh School of Medicine, Pittsburgh, PA USA; 9 Department of Otolaryngology, University of Pittsburgh School of Medicine, Pittsburgh, PA USA

**Keywords:** Head and neck cancer, Tumour immunology

## Abstract

**Background:**

Head and neck cancers (HNSCC) are highly immunosuppressive. Plasma-derived exosomes of HNSCC patients carry immunomodulatory molecules, and their cargo correlates with clinical parameters. Here, we evaluated the exosomal molecular profile for early detection of treatment failure in locally advanced HNSCC patients treated with conventional therapy.

**Methods:**

Plasma from 17 HNSCC patients was collected before, during, and after treatment by surgery with adjuvant (chemo)radiation and at recurrence. Exosomes were isolated by size-exclusion chromatography. Total exosomal protein (TEP) was used to estimate exosome load and on-bead flow cytometry to evaluate relative fluorescence intensity (RFI) of tumour-associated and immunoregulatory proteins on exosomes. Exosomal effects on the activity of and adenosine production by T cells was assessed by flow cytometry and mass spectrometry.

**Results:**

TEP and the ratio of tumour-/immune-cell-derived exosomes varied during and after therapy with an overall decrease in the tumour-free follow-up but an increase at recurrence. RFI values of immunoregulatory proteins on exosomes, their ability for T cell inhibition and adenosine production changed during and after therapy. PD-L1 was the earliest discriminator for treatment failure and disease-free survival.

**Conclusions:**

Monitoring of plasma exosomes during therapy represents a promising opportunity for early detection of treatment failure and risk stratification to delay/avoid recurrence.

## Background

With ~900,000 diagnosed cases and around 450,000 deaths per year [1], head and neck squamous cell carcinoma (HNSCC) belongs to the seven most common cancers worldwide. Most patients present with locally advanced disease for which multimodal curative treatment is available, consisting of surgery followed by risk-adapted adjuvant (chemo)radiotherapy ((C)RT) or primary CRT [2]. Yet, disease outcome remains poor, largely due to loco-regional recurrence or distant metastasis.

HNSCC is characterised by profound immune suppression. Among various immunosuppressive factors present in the tumour microenvironment (TME), exosomes have been of special interest [3, 4]. Exosomes are the smallest (30–150 nm) extracellular vesicles (EVs) that are released by all cell types and mediate intercellular communication in physiological and pathological settings [5, 6]. Due to their distinctive biogenesis in the late endosomes, their unique molecular cargo, and the ability to freely circulate in all body fluids, exosomes are of great interest as potential components of non-invasive liquid biopsy [7–9]. Plasma of HNSCC patients is highly enriched in tumour-cell-derived exosomes, which carry immunosuppressive proteins and are able to alter immune cell functions [10]. Our previous studies had emphasised the potential of exosomes isolated from patients blood plasma as liquid biomarkers for disease stage, tumour activity and progression [11–15]. Further, we showed that palliative therapeutic interventions change the exosome cargo and their potential to induce epithelial-to-mesenchymal transition [16]. Moreover, the exosome cargo was used to discriminate between responders and non-responders after treatment with cetuximab, ipilimumab and radiation therapy [17]. These results support the use of exosomes as a forecast for therapy outcome. However, no studies were conducted so far in HNSCC patients receiving conventional therapy.

In the present pilot study, we analyze the potential of circulating exosomes as early detectors of treatment success in a cohort of locally advanced HNSCC patients undergoing conventional treatment. By examining plasma-derived exosomes from defined time-points before, during, and after therapy, we aimed to determine the change in the molecular profile of exosomes during and after therapy, which enables the earliest prediction of recurrence.

## Methods

### Patients

As part of the non-interventional, longitudinal IRECT study (Immune Response Evaluation to Curative Conventional Therapy; NCT03053661), peripheral blood samples were obtained prospectively from 17 newly diagnosed HNSCC patients with histologically confirmed tumours who were treated at the Head and Neck Cancer Center of the Comprehensive Cancer Center Ulm (CCCU) from 2013 to 2015 as previously described [18, 19]. Blood sample collection was approved by the Ethics Committee of the University of Ulm (#222/13) and each patient provided informed consent.

In accordance with international treatment guidelines and by recommendation of the interdisciplinary tumour board at the CCCU, patients were treated with primary surgery, surgery with adjuvant radiotherapy (RT) or surgery with adjuvant chemoradiotherapy (CRT). Blood samples were taken at defined time-points before, during, and after curative treatment (Supplementary Fig. S1). They were collected in citrate tubes and centrifuged at 1000 × *g* for 10 min followed by 2500 × *g* for 10 min. The resulting plasma specimens were stored in aliquots at −80 °C. Table 1 provides the clinicopathological characteristics of the patients included in this study.

**Table 1:** Clinicopathological data of HNSCC patients enrolled in this study

Characteristics	Patients (*n* = 17)	
	*n*	%
Age (years)		
≤60	9	53
>60	8	47
(Range: 53–73)		
Gender		
Male	16	94
Female	1	6
Primary tumour site		
Oral cavity	3	17
Oropharynx	10	59
HPV positive	7	70
HPV negative	3	30
Hypopharynx	2	12
Larynx	2	12
Tumour stage		
T1	5	29.5
T2	6	35
T3	5	29.5
T4	1	6
Nodal status		
N0	7	41
N+	10	59
Distant metastasis		
M0	17	100
Therapy		
Surgery	4	24
Surgery + adj. CRT	6	35
Surgery + adj. RT	7	41
Recurrence		
Yes	5	29.5
No	12	70.5

*HPV* Human papillomavirus, *CRT* chemoradiotherapy, *RT* radiotherapy.

### Exosome isolation by size-exclusion chromatography (SEC)

For exosome isolation, frozen plasma was processed by size-exclusion chromatography (SEC) as previously described [20]. Briefly, freshly thawed plasma was sequentially centrifuged at 2000 × *g* for 10 min at room temperature (RT) and 10,000 × *g* for 30 min at 4 °C to remove cell debris and larger vesicles, followed by filtration through 0.22 μm syringe-filters (Millipore, Burlington, MA, USA, SLGPO33RB). Aliquots (1 mL) of plasma were loaded on SEC columns and eluted with PBS. Sequential 1 mL fraction #4 highly enriched in exosomes [20] was collected and used for further analysis.

### BCA and exosome concentration

Total exosomal protein (TEP) concentration was measured by Pierce BCA Protein Assay (ThermoFisher Scientific, Waltham, MA, USA, 23225) according to the manufacturer’s protocol. Exosomes were concentrated using 100 kDa cut-off centrifugal filters (Millipore, UFC5100BK). For on-bead flow cytometry, 10 µg of exosomes in 100 µL PBS were used; for western blot, 20 µg of exosomes in 40 µL PBS were used and for functional assays, 10 µg of exosomes in 50 µl or 100 µl were used, as described in the respective methods section.

### Characterisation of exosomes

The methods for exosome characterisation are in line with the minimal information for studies of extracellular vesicles (MISEV) 2018 guidelines for the definition of EVs [21], and are routinely performed as described in detail in our previous publication [12] (EV-TRACK ID: EV200068).

### Immune capture and on-bead flow cytometry of exosomes

Exosomes were captured on ExoCap Streptavidin magnetic beads (MBL Life Science, Woburn, MA, USA, MEX-SA) as previously described [12, 14, 15, 17]. Briefly, exosomes (10 µg in 100 µL PBS) were incubated for 2 h at RT on a shaker with biotin-labeled anti-CD63 (BioLegend, San Diego, CA, USA, 353018, RRID:AB_2561676) adjusted to a concentration of 1 µg in 100 µL PBS. Next, 10 µl of beads were added and samples were again incubated for 2 h at RT on a shaker. The uncaptured fraction was removed and samples were washed using a magnetic rack.

For antigen surface detection by on-bead flow cytometry, the bead/anti-CD63/exosome complexes were incubated with fluorophore-conjugated antibodies for 1 h at RT on a shaker. The following detection antibodies and appropriate isotype controls were used: anti-PD-L1-PE (12-5983-42, RRID:AB_11042286), anti-OX40-PE (12-1347-42, RRID:AB_10668832), anti-CD3-PE (12-0037-42, RRID:AB_1272078), anti-mouse-IgG1-PE (12-4714-42, RRID: AB_1944423) and anti-mouse-IgG2a-PE (12-4724-82, RRID:AB_470064) from eBioscience (San Diego, CA, USA); anti-CTLA-4-APC (349908, RRID:AB_10679122) and anti-mouse-IgG1-APC (400122, RRID:AB_326443) from BioLegend; anti-OX40-L-APC (FAB10541A, RRID:AB_10642181), anti-CD44v3-APC (FAB5088A, RRID:AB_2076584), anti-mouse-IgG1-APC (IC002A, RRID:AB_357239) and anti-mouse-IgG2b-APC (IC0041A, RRID:AB_357246) from R&D Systems (Minneapolis, MN, USA). Next, the stained complexes were washed twice with PBS and resuspended in 300 µl PBS for flow cytometry. Detection was performed using a Gallios flow cytometer with Kaluza 1.0 software (Beckman Coulter, Brea, CA, USA, RRID:SCR_016182). Samples were run for 2 min and 10,000 events were acquired. Gates were set in the bead fraction visible in the forward/sideward light scatter. Data are presented as relative fluorescent intensity (RFI), which equals the mean fluorescence intensity of the stained sample divided by the mean fluorescence intensity of the isotype control. The CD44v3/CD3 ratio was calculated for each sample by dividing its RFI value for CD44v3 by the mean RFI for CD3.

### Suppression of CD69 expression and induction of CD8^+^ T cell apoptosis by exosomes

Peripheral blood mononuclear cells (PBMCs) were harvested from healthy donor’s buffy coats (obtained from DRK Ulm, Helmholtzstraße 10, 89081 Ulm) using density gradient centrifugation with Biocoll separation solution (Bio&SELL, Feucht/Nürnberg, Germany, L6115) and Leukosep tubes (Greiner Bio-One, Kremsmünster, Österreich, 227290). CD8^+^ T cells were isolated from PBMCs by negative selection using the human CD8^+^ T Cell Isolation Kit (Miltenyi Biotec, Bergisch Gladbach, Germany, 130-096-495) according to manufacturer’s instruction. CD8^+^ T cells (1.3 × 10^6^/mL) were activated with 25 µl/mL ImmunoCult™ Human CD3/CD28 T Cell Activator (Stemcell Technologies, Vancouver, Canada, 10971) and 150 U/mL recombinant human IL2 (R&D Systems, 202-IL-010) in RPMI (21875-034) supplemented with 10% exosome-depleted FBS (both Gibco, Carlsbad, CA, USA, A2720801) and 1% penicillin-streptomycin. One hundred fifty microlitres (200,000 cells) of activated T cells were seeded in flat-bottom 96-well plates and incubated at 37 °C for 6 h (CD69 induction) or 16 h (apoptosis). Exosomes (10 µg in 50 µl PBS) isolated from plasma of HNSCC patients were added and co-cultures were incubated for 16 h (CD69 induction) or 6 h (apoptosis). As control, 50 µl of PBS were added instead of exosomes. Expression levels of CD69 and apoptosis of activated T cells were measured on a Gallios flow cytometer using 20 µl of CD69-FITC antibody (BD, Franklin Lakes, NJ, USA, 555530, RRID:AB_395915) and Annexin V-FITC Apoptosis Staining/Detection Kit (Abcam, Cambridge, UK, ab14085). Both protocols were previously described [10, 11].

### Adenosine production by exosomes or CD4^+^ CD39^+^ T cells co-incubated with exosomes

Healthy donor’s PBMCs were harvested as described above. CD4^+^ CD39^+^ T cells were isolated from PBMCs by negative selection of CD4^+^ cells using the human CD4^+^ T Cell Isolation Kit (130-096-533) followed by positive selection of CD39^+^ cells using a biotinylated anti-CD39 antibody (130-093-505, RRID:AB_1036212) and anti-biotin MicroBeads (130-090-485, RRID:AB_244365, all from Miltenyi Biotec) according to manufacturer’s instruction and as described previously [13, 22].

Freshly isolated CD4^+^ CD39^+^ T cells (25,000 cells in 50 µl PBS) were incubated with exosomes (10 µg in 50 µl PBS) isolated from plasma of HNSCC patients and 20 µM adenosine triphosphate (ATP) for 1 h at 37 °C. Additionally, exosomes alone (10 µg in 100 µl PBS) were incubated with 20 µM ATP for 1 h at 37 °C. Samples were centrifuged at 6000 × *g* for 2 min, supernatants were boiled for 2 min at 95 °C and stored at −80 °C until further processing. Concentrations of 5‘ adenosine monophosphate (AMP) and adenosine, as well as their degradation product hypoxanthine, were measured by mass spectrometry, as described previously [22]. As controls, T cells incubated with ATP, exosomes incubated without ATP, and ATP in PBS only were used.

### Statistics

Statistcal analysis was performed using GraphPad Prism (version 8.4.3, GraphPad Software, San Diego, CA, RRID:SCR_002798). Data are indicated as box-and-whisker blots with the line representing the median, the box showing the interquartile range (25–75%) and the whiskers indicating the range. Comparisons between time-points were analyzed using Wilcoxon test for related samples and Mann–Whitney test for independent samples. *P* < 0.05 was considered statistically significant. For determination of the exoPD-L1 status at different time-points, the median of the respective PD-L1 RFI values was used as cut-off. RFIs < median were considered exoPD-L1 low and RFIs > median exoPD-L1 high. Disease-free survival (DFS) was evaluated by the Kaplan–Meier method and differences between survival curves were assessed using the log-rank test. The interval of DFS was measured from the date of diagnosis to the date of recurrence (*n* = 5) or death (*n* = 1).

## Results

### Study population

The clinicopathological characteristics of the HNSCC patients whose plasma was used for exosome isolation are listed in Table 1. At the time-point of diagnosis, the mean age was 60 years with a range from 53 to 73 years. Most patients (94%) were male. The primary tumour was located in the oral cavity (18%), pharynx (70%) or larynx (12%). Among the 17 patients, 35% presented with advanced (T3/4) tumour stage and 59% had lymph node metastasis. Patients were treated with primary surgery (4/17), surgery and adjuvant CRT (6/17) or surgery and adjuvant RT (7/17). All patients were followed for disease progression, and the median follow-up was 67 months (range 25–81 months). The time-point of follow-up refers to 12 months after therapy in non-recurrent patients or to the last available time-point before diagnosis of recurrence. One patient had a residual tumour after incomplete therapy due to patient decision, while four patients experienced recurrence in the follow-up period (>11 months after treatment termination) as determined by clinical evaluation and annual imaging scans. Three of the five recurrent patients were treated with primary surgery and one patient each with surgery and adjuvant CRT or RT. 12 patients were disease free (median follow-up 68 months).

### Characterisation of exosomes

Exosomes isolated from plasma of HNSCC patients by SEC were evaluated for morphology by TEM, for size by NTA, and for their protein composition by western blot analysis. Isolated exosomes showed vesicular morphology (Supplementary Fig. S2A) and had a median diameter of 79 nm (Supplementary Fig. S2B). Further, exosome preparations were confirmed to be positive for the endosomal marker TSG101 and other vesicle-associated proteins such as CD9 and CD63 (Supplementary Fig. S2C). The non-exosomal marker Grp94 and the apolipoprotein (ApoA1) were not detected. The described methods for confirmation of exosome nomenclature follow the MISEV 2018 guidelines [21] and are routinely followed as described in our previous publications [10, 12].

### Changes in tumour- and T-cell-derived exosomes and their protein load during and after therapy

**Fig. 1:**
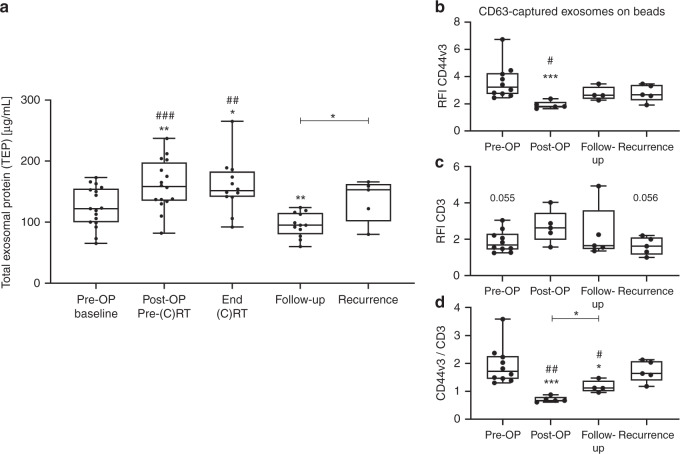
Total exosomal protein (TEP), CD44v3 and CD3 values on exosomes isolated from plasma of HNSCC patients. **a** TEP concentrations of the exosome fraction obtained from plasma of *n* = 17 head and neck squamous cell carcinoma (HNSCC) patients at defined time-points was measured by bicinchoninic acid (BCA) assay. Results are plotted as box-and-whisker blots representing the median value, the 25th and 75th quartiles and the range. Wilcoxon related samples tests were applied to each compare pre-OP, post-OP, end (C)RT and follow-up. Mann–Whitney test was applied to compare recurrence with follow-up. *, ** or ^##^ and ^###^ correspond to *p* ≤ 0.05, *p* ≤ 0.01 and *p* ≤ 0.001, respectively. Asterisks and hashtags above boxes indicate the significance compared to baseline and follow-up, respectively. (C)RT = (chemo)radiotherapy. **b**–**d** Exosomes from HNSCC patients were captured using biotinylated CD63-antibodies and stained with fluorochrome-conjugated antibodies against CD44v3 (**b**) and CD3 (**c**). Surface values as determined by on-bead flow cytometry are shown as relative fluorescence intensity (RFI) compared to an appropriate isotype control. In **d**, the ratio between CD44v3^+^ (Tumour-derived) and CD3^+^ (T-cell-derived) exosomes is shown. Results are plotted as box-and-whisker blots representing the median value, the 25th and 75th quartiles and the range from *n* = 10 (pre-OP), *n* = 5 (post-OP, non-recurrence, recurrence) HNSCC patients. *P-*values were determined by Mann–Whitney test, with * or ^#^, ^##^, *** corresponding to *p* ≤ 0.05, *p* ≤ 0.01 and *p* ≤ 0.001, respectively. Asterisks, hashtags or values above boxes indicate the significance compared to following: **b** asterisks pre-OP, hashtag non-recurrence and recurrence, **c** values post-OP, **d** asterisks pre-OP, hashtags recurrence.

Total exosomal protein (TEP) was measured to estimate the exosome load. Compared to baseline, TEP showed a significant increase after surgery and at the end of (C)RT (Fig. 1a). Overall, a significant decrease in TEP was visible during the disease-free follow-up period (Fig. 1a). At the time of recurrence, TEP was significantly increased and was as high as at the end of (C)RT (Fig. 1a).

Both tumour and T-cell-derived exosomes are known to be important modulators of the tumour microenvironment [9]. To detect relative content of tumour-derived CD44v3^+^ exosomes and of T-cell-derived (CD3^+^) exosomes among the total exosomes isolated from plasma, on-bead flow cytometry was used. The RFI values for CD44v3^+^ exosomes were significantly decreased after surgery (post-OP) and significantly increased both during the disease-free follow-up and at the time of recurrence (Fig. 1b). The RFI values for CD3^+^ exosomes increased after surgery and decreased at the time of recurrence, albeit these changes were not significant (Fig. 1c). Importantly, the ratio of tumour- and T-cell-derived exosomes (CD44v3^+^/CD3^+^) significantly decreased after surgery (Fig. 1d), indicating a reduced tumour load. This ratio significantly increased during the follow-up period in both responders and non-responders to therapy. However, non-responders had a significantly higher ratio compared to responders (Fig. 1d), indicating an increase in tumour-derived exosomes.

### Changes in immune checkpoints carried by exosomes during and after therapy

**Fig. 2:**
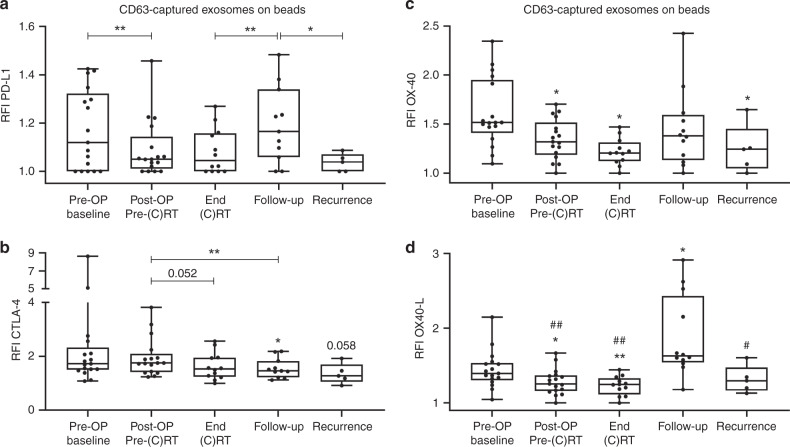
Surface values of immune checkpoints on exosomes isolated from plasma of HNSCC patients. Exosomes from *n* = 17 HNSCC patients obtained at defined time-points were captured using biotinylated CD63-antibodies and stained with fluorochrome-conjugated antibodies against PD-L1 (**a**), CTLA-4 (**b**), OX40 (**c**) and OX40-L (**d**). Surface values as determined by on-bead flow cytometry are shown as RFI compared to an appropriate isotype control. Results are plotted as box-and-whisker blots representing the median value, the 25th and 75th quartiles and the range. Wilcoxon related samples tests were applied to each compare pre-OP, post-OP, end (C)RT and follow-up. Mann–Whitney test was applied to compare recurrence with follow-up. * or # and ** or ^##^ correspond to *p* ≤ 0.05 and *p* ≤ 0.01, respectively. Asterisks and values above boxes indicate the significance compared to pre-OP. Hashtags above boxes indicate the significance compared to follow-up. **c** RT (chemo)radiotherapy

On-bead flow cytometry was used to determine RFI values for the co-inhibitory immune checkpoints, PD-L1 and CTLA-4, and the co-stimulatory immune checkpoints, OX40 and OX40-L, carried by exosomes. The RFI values for PD-L1 were significantly reduced after surgery (Fig. 2a). During the disease-free follow-up period, the RFI values for PD-L1 significantly increased compared to the RFI values at the end of (C)RT. At the time of recurrence, RFI values for PD-L1 were significantly reduced. Baseline RFI values for CTLA-4 were high in individual patients and significantly declined during the follow-up period compared to pre- and post-surgery (Fig. 2b). Compared to baseline, RFI values for OX40 were significantly reduced after surgery, at the end of (C)RT and at the time of recurrence (Fig. 2c). Only during the disease-free follow-up period, RFI values for OX40 were increased but not significantly. Similar to OX40, RFI values for OX40-L significantly decreased after surgery and at the end of (C)RT (Fig. 2d). During the disease-free follow-up period, RFI values for OX40-L significantly increased, showing the highest values compared to all other time-points. At the time of recurrence, RFI values for OX40-L significantly decreased.

### PD-L1 as the earliest discriminator for treatment failure and disease-free survival

**Fig. 3:**
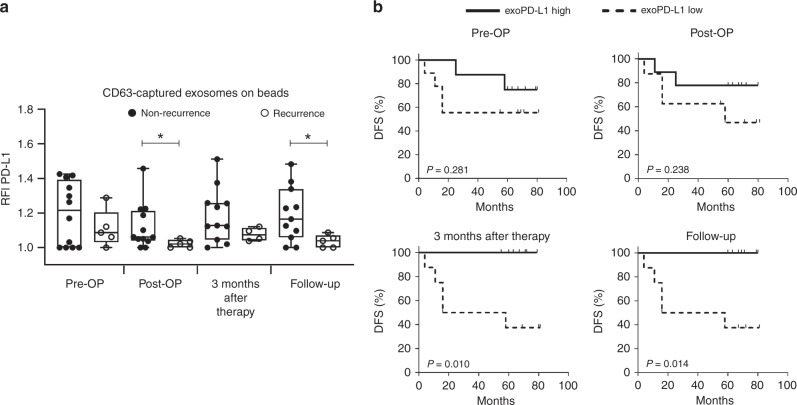
PD-L1 on exosomes isolated from plasma of HNSCC patients. **a** Exosomes from *n* = 12 successfully treated and *n* = 5 recurrent HNSCC patients were captured using biotinylated CD63-antibodies and stained with fluorochrome-conjugated antibodies against PD-L1. Surface values as determined by on-bead flow cytometry are shown as RFI compared to an appropriate isotype control. Results are plotted as box-and-whisker blots representing the median value, the 25th and 75th quartiles and the range. *P*-values were determined by Mann–Whitney test, with * corresponding to *p* ≤ 0.05. **b** Kaplan–Meier curves with log-rank test for disease-free survival (DFS) of *n* = 17 HNSCC patients grouped according their exosomal PD-L1 (exoPD-L1) values. ExoPD-L1 status was determined at different time-points using the median as cut-off. Solid line indicates high exoPD-L1 and dashed line low exoPD-L1.

While RFI values for all the assessed markers were decreased at the time of recurrence, the RFI values for PD-L1 measured post-OP identified it as the earliest time-point to discriminate between successfully treated and recurrent patients. After surgery (post-OP), the RFI values for PD-L1 in patients who had no recurrence were significantly higher than the values for patients whose tumours recurred (Fig. 3). At the time of relapse, patients with recurrent disease had significantly reduced RFI values for PD-L1 compared to patients with no recurrence during the follow-up period (Fig. 3a). The association between RFI values for PD-L1 (exoPD-L1) and disease-free survival (DFS) was evaluated in Kaplan–Meier plots (Fig. 3b). The time interval until relapse was lower in patients with exoPD-L1 low status at all time-points with significant evidence of a difference in DFS at 3 months after therapy and in the follow-up period. Recurrent patients exhibited heterogeneity with regard to tumour site and stage with no correlation to the exoPD-L1 status. Although RFI values of other markers were significantly reduced at the time of recurrence, none other marker than PD-L1 could consistently discriminate between successfully treated and recurrent patients at an earlier time-point and at the follow-up time-point (Supplementary Fig. S3). Although CTLA-4 RFI values were significantly elevated in recurrent patients at 3 months after therapy, this difference was not observed in the long-term follow-up.

### Therapy-induced modulation of adenosine production by exosomes

In previous studies, we reported that exosomes independently produce immunosuppressive adenosine and induce adenosine production in CD4^+^CD39^+^ T cells, a subset of antigen-experienced and regulatory T cells [10, 13, 22, 23]. To evaluate effects of therapy on exosome adenosine production, we co-incubated exosomes alone or with CD4^+^CD39^+^ T cells isolated from healthy donors in the presence of exogenous ATP. Following, the conversion of ATP to 5’-AMP and adenosine was measured by mass spectrometry.

**Fig. 4:**
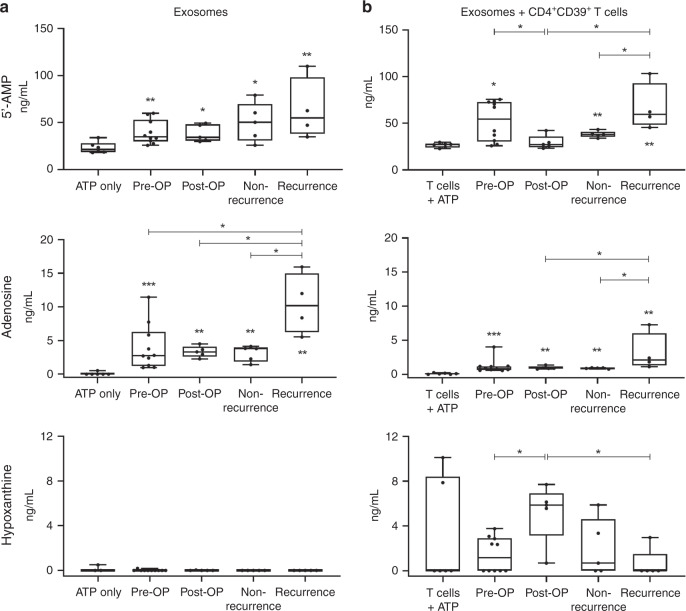
Nucleoside production of exosomes and CD4^+^ CD39^+^ T cells co-cultured with exosomes. 5’-AMP, adenosine and hypoxanthine levels were measured by mass spectrometry upon incubating exosomes alone (**a**) or exosomes plus CD4^+^ CD39^+^ T cells (**b**) with exogenous ATP. ATP only (**a**) and CD4^+^ CD39^+^ T cells with ATP (**b**) were included as controls. All results are plotted as box-and-whisker blots representing the median value, the 25th and 75th quartiles and the range from *n* = 6 replicates (controls), *n* = 10 (pre-OP), *n* = 5 (post-OP, non-recurrence, recurrence) HNSCC patients. All *p-*values were determined by Mann–Whitney test, with *, ** and *** corresponding to *p* ≤ 0.05, *p* ≤ 0.01 and *p* ≤ 0.0001, respectively. Asterisks above or below boxes indicate the significance compared to the control.

Exosomes from all time-points induced conversion of ATP to 5’-AMP and further to adenosine (Fig. 4a). While serially collected exosomes at various time-points showed no differential effects of therapy on 5’-AMP production (Fig. 4a, top), significantly increased adenosine production was observed in exosomes of patients with recurrence (Fig. 4a, middle panel).

In the presence of exosomes, CD4^+^CD39^+^ T cells showed a significant upregulation of 5’-AMP and adenosine production (Fig. 4b). Exosomes from plasma harvested before surgery induced a high level of 5’-AMP in CD4^+^CD39^+^ T cells, which significantly decreased with exosomes harvested post-OP (Fig. 4b, top). The comparison of exosomes from patients with or without recurrence, showed significantly greater ability to induce 5’-AMP and adenosine production in CD4^+^CD39^+^ T cells by exosomes of patients with recurrence (Fig. 4b, top and middle**)**. In the presence of post-OP exosomes, which did not induce adenosine in CD4^+^CD39^+^ T cells, hypoxanthine levels produced by CD4^+^CD39^+^ T cells were significantly elevated (Fig. 4b, bottom). This finding indicates that post-OP exosomes effectively induced adenosine degradation to hypoxanthine in CD4^+^CD39^+^ T cells, thus decreasing adenosine levels and adenosine-associated immune suppression mediated by these cells.

### Therapy-induced modulation of exosome-mediated immunosuppression

**Fig. 5:**
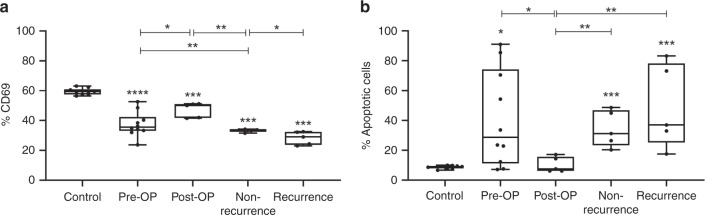
CD69 expression and apoptosis of activated CD8^+^ T cells. Activated CD8^+^ T cells were co-incubated with exosomes isolated from plasma of HNSCC patients at defined time-points. Activation (**a**) and apoptosis (**b**) of CD8^+^ T cells were determined by flow cytometry using fluorochrome-conjugated antibodies against CD69 and annexin/propidium iodide staining, respectively. Results are shown as percentage of CD69 positive cells (**a**) and annexin positive cells (**b**). All results are plotted as box-and-whisker blots representing the median value, the 25th and 75th quartiles and the range from *n* = 10 replicates (control), *n* = 10 (pre-OP), *n* = 5 (post-OP, non-recurrence, recurrence) HNSCC patients. All *p-*values were determined by Mann–Whitney test, with *, **, *** and **** corresponding to *p* ≤ 0.05, *p* ≤ 0.01, *p* ≤ 0.001 and *p* ≤ 0.0001, respectively. Asterisks above or below boxes indicate the significance compared to the control.

To evaluate exosome-mediated immunosuppressive effects on cytotoxic lymphocytes, primary human CD8^+^ T cells were co-incubated with the exosomes serially harvested at various time-points during therapy. Following co-incubation, activation (CD69 expression) and apoptosis of CD8^+^ T cells were assessed by flow cytometry. Untreated T cells had high CD69 expression levels (~60%) (Fig. 5a). CD69 expression was significantly decreased upon co-incubation with exosomes derived from plasma collected at all time-points, although differences between the single time-points were observed. Exosomes of pre-OP patients strongly reduced CD69 expression of activated T cells (down to ~35%). T cells co-incubated with post-OP exosomes showed significantly higher CD69 expression levels (~45%). Thus, post-OP exosomes had reduced inhibitory effects on CD8^+^ T cells compared to pre-OP exosomes. Significant inhibition of CD8^+^ T cell activation by exosomes from plasma of successfully treated and recurrent patients was observed, but because of limited specimen numbers in the recurrent patient group, the significance of greater inhibition of CD69 expression in exosomes from recurrent patients remains unclear.

Apoptosis of activated CD8^+^ T cells assessed by annexin/propidium iodide staining and flow cytometry showed that compared to untreated control T cells (~10% apoptosis), T cells co-incubated with exosomes obtained from pre-OP plasma had significantly higher apoptosis (~40%) (Fig. 5b). Exosomes from the post-OP plasma mediated almost no apoptosis, similar to controls. In comparison to apoptosis induced by post-OP exosomes, those obtained from plasma of responders or non-responders induced significantly more apoptosis, with no significant difference between them.

## Discussion

There is an unmet need for discovery of non-invasive or minimally invasive biomarkers for diagnosis, therapy monitoring, and follow-up in cancer patients. The early detection of treatment failure or disease recurrence is of special significance, as it offers an opportunity for a timely change in therapy and potential improvement in prognosis. This pilot study in a cohort of locally advanced HNSCC patients treated with surgery and adjuvant (C)RT was initiated with the goal of evaluating plasma exosomes as potential determinants of early failure of oncological therapy and recurrence. We chose to examine plasma exosomes based on previous studies, which showed that the EV volume in plasma of HNSCC patients and their molecular content associated with disease activity, stage and lymph node involvement [10, 11]. We also reported that pre-therapy levels of EVs and changes in the vesicle phenotypic characteristics during therapy combining cetuximab, ipilimumab and intensity-modulated radiation therapy (IMRT) predicted response to therapy, demonstrating that EVs might serve as promising predictive biomarkers in patients with HNSCC [17]. In these studies, a subset of small EVs, referred to as exosomes and defined by their origin, size and molecular cargo was the investigated target, with tumour-derived exosomes emerging as the highly informative vesicle subset.

Here, we monitored exosomes in serially collected plasma of HNSCC patients treated with curative surgical therapy and showed significant changes in TEP levels, the ratio of tumour-/immune-cell-derived exosomes, profiles of surface proteins and immunosuppressive activity of exosomes during therapy and the follow-up period. Since plasma EVs are a heterogenous population of vesicles produced by all cells, the identification of exosomes enriched in CD44v3 protein, an antigen overexpressed on HNSCC cells, facilitated interrogation of tumour-derived exosomes (TEX) as surrogates of the tumour [14]. Similarly, on-bead flow cytometry for CD3^+^ (i.e. T-cell-derived) exosomes provided data informing about the immune-related changes in response to therapies. We serially monitored changes in exosome profiles or functions from the pre-therapy baseline to the time at which five patients developed recurrence (mean 21 months later). We examined and compared changes induced by therapies and occurring during the follow-up period in patients who were successfully treated and those who developed recurrence. We searched for the significant change in the exosome profiles that would allow for an early discrimination of patients who remain disease free from those who experience recurrence. Remarkably, despite the small number of patients with recurrence, serial profiling of plasma exosomes indicated that PD-L1^+^ exosomes were the highly significant and most consistent early indicator of successful treatment in HNSCC patients enrolled in the study. The RFI values for PD-L1 on exosomes at the pre-surgery baseline were not significantly different in successfully treated vs recurrence-developing patients. After surgery, these RFI PD-L1 values decreased significantly in later recurrent patients and remained low until recurrence. Possible reasons for the lower exosomal PD-L1 values at the time-point of recurrence compared to the pre-therapuetic situation may be a reduced immune infiltrate in the recurrent tumour [24, 25] or a lower grade of differentiation of the usually more aggressive recurrent tumour compared to the primary, treatment-naïve tumour [26–29]. In contrast, in successfully treated patients, the RFI values for PD-L1 on exosomes remained elevated throughout the follow-up period. The results stratified patients into two groups for DFS (*p* = 0.010) and were somewhat counterintuitive, as patients with high PD-L1 on exosomes had a better outcome.

PD-L1 is an immunosuppressive ligand, which is usually associated with immune evasion. However, PD-L1 expression is not necessarily a sign of immune evasion but can also reflect an active endogenous anti-tumour immune response resulting from production of pro-inflammatory IFNγ in the TME [30–32]. Despite extensive studies, the prognostic impact of PD-L1 expression in the tumour remains inconsistent for HNSCC, with some studies associating high PD-L1 expression to good prognosis and others linking high PD-L1 expression to bad prognosis [32–37]. However, PD-L1 expression based solely on results of immunohistochemistry in cancer cells might not be a robust prognostic indicator. Tumour heterogeneity, space and dynamics of PD-L1 expression by both tumour and immune cells, treatment regimens as well as time and quality of tumour biopsy may adversely influence prognostic significance of PD-L1 expression [32, 35, 36]. A more reliable and consistent measure of the PD-L1 status than the currently used PD-L1 combined positive score (CPS) is an unmet need. In patients with recurrent or metastatic HNSCC, the CPS of the tumour tissue is performed to identify patients who may benefit from pembrolizumab with or without platinum-based chemotherapy [38]. The guidelines do not specify whether to use the primary or recurrent tumour for CPS determination. In the absence of a second biopsy after initial treatment, CPS may be obtained from the treatmen-naïve tumour, which clearly does not reflect the current PD-L1 status of the recurrent tumour. For this and similar clinical situations, exosome-based evaluation of the patients’ PD-L1 status could be an attractive solution. For the future, the exoPD-L1 status seems as a promising, non-invasive, antibody based and thus highly specific approach that could be considered as “liquid CPS”. Also, evaluation of PD-L1 on exosomes measures both the tumour-derived and immune-cell-derived PD-L1. Recently, PD-L1 on circulating exosomes from melanoma and lung cancer patients was reported to be a better biomarker than PD-L1 expression in tumour biopsies [39, 40]. Further, exosomal PD-L1 predicted melanoma patients’ response to immunotherapy, with non-responders showing an increase of PD-L1 [39, 41]. However, to our knowledge, this is the first study investigating the association of PD-L1 and conventional therapies. While our results associate high PD-L1 on exosomes with improved DFS in HNSCC patients treated with conventional therapies, an association between exosomal PD-L1 and patients’ response to immunotherapy in HNSCC remains to be determined in future studies of patients treated with immune checkpoint inhibitors since no current studies are available in this regard.

We have shown previously that, in addition to PD-L1, plasma-derived exosomes carry a variety of other immune inhibitory and stimulatory molecules, including CTLA-4, OX40 and OX40-L, which can be detected and quantified by on-bead flow cytometry [10, 11, 15, 42]. Therefore, we evaluated changes in the RFI values of these proteins on the surface of plasma exosomes after surgery and adjuvant (C)RT. Somewhat unexpectedly, no informative early changes in these other checkpoint proteins were noted, although a significant decrease in co-stimulatory proteins after (C)RT, recovery in the follow-up period and significant decrease in patients with recurrence were observed. As previously reported, disease progression was associated with a significant decrease in exosomes enriched in co-stimulatory proteins [15, 17]. However, the here described changes of immune checkpoint molecules on exosomes were not correlative to immune checkpoint expression on immune cells from corresponding HNSCC patients after (C)RT [18], which might be due to the investigation of the total exosome population in our study versus specific immune cell subpopulations in the other study. From our data, we can not distinguish from which exosome subpopulation the observed changes of immune checkpoint molecules arise as the here analyzed total exosome population resembles changes of the whole TME.

This study also investigated changes in plasma TEP levels during therapy based on the rationale that TEP levels might serve as an indicator of disease activity and treatment success. Surprisingly, TEP levels at the pre-OP baseline were not predictive of successful surgery/(C)RT in patients with HNSCC as previously reported for the naïve patients with non-small-cell lung carcinoma treated with chemotherapy [43]. Also, an increase in TEP levels was observed after surgery which was unexpected in view of the data reported for patients with oral squamous cell carcinoma [44]. However, while TEP levels increased post-OP, RFI values for CD44v3^+^ exosomes decreased, suggesting a lower content of TEX in post-OP vesicles. Also, RFI values for CD3^+^ exosomes increased post-OP suggesting that the increase in TEP levels was largely due to exosomes produced by T lymphocytes possibly as a sign of an inflammatory response. Importantly, TEP levels remained elevated during/after (C)RT, but then declined and remained low in the follow-up period, except in patients who experienced recurrence. Thus, TEP levels remain a correlate of disease progression/recurrence, and pending further evaluation, might qualify as a measure of outcome in patients with HNSCC treated with (C)RT.

Plasma-derived exosomes in HNSCC patients represent a potpourri of TEX and non-malignant cell-derived exosomes (non-TEX), which are mainly derived from immune cells [14, 15, 45]. The flow-based analysis of CD44v3^+^ TEX and CD3^+^ non-TEX accurately reflected expected post-OP changes in the tumour burden (decrease) and T lymphocyte activity (increase). These findings suggest that plasma exosomes might accurately inform about the success of a surgery, especially when considered as the ratio of TEX/non-TEX, which after a significant post-OP decline, climbed up to the high pre-therapy values in patients who experienced recurrence. It appears that monitoring of TEX/non-TEX ratios during and post-therapy can provide useful non-invasive information about the success of (C)RT in patients with advanced/metastatic HNSCC, as also previously reported for HNSCC patients treated with immuno-chemoradiotherapy [17]. The ratio might be a late rather than early discriminator of response to (C)RT, but it may be able to identify patients who are likely to recur during the follow-up period, thus distinguishing responders from non-responders. Additional studies are needed to confirm this promising evidence for the role of the TEX/non-TEX ratio in predicting disease recurrence.

Finally, functional in vitro studies with the plasma-derived exosomes serially harvested during therapy and co-incubated with primary T cells confirmed previously reported immunoinhibitory activity of circulating exosomes in HNSCC patients [10, 45]. Not surprisingly, all the immunosuppressive functions measured (inhibition of T cell activation, effector T cell apoptosis, adenosine production and induction of CD4^+^CD39^+^ T-cell-mediated suppression), were ameliorated after surgery relative to pre-surgery measures. This fact alone testifies to the accuracy of exosomes in reflecting the overall immune status of cancer patients and changes in the immune responses that occur during therapy. Notably, exosome-mediated immunosuppression was elevated, approaching the values measured at baseline, in patients with recurrence compared to patients who remained disease free in the follow-up period. Thus, exosome immunoregulatory functions appear to be useful in forecasting improvements or declines in the course of the disease in patients with HNSCC as previously reported [17]. Since PD-L1 presence on plasma exosomes contributes to their immunoregulatory activity, and exosomal PD-L1 appears to be an early indicator of response to (C)RT, it is reasonable to conclude that functions of plasma-derived exosomes represent promising predictive biomarkers that in the future might replace labor-intensive and complex immune monitoring performed with harvested immune cells.

Overall, exosomes, and especially exosome subsets enriched in TEX, that are harvested from HNSCC patients’ plasma at different time-points prior to, during and after therapy emerge as promising non-invasive biomarkers able to discriminate early in the course of treatment between patients who can be cured by surgery/(C)RT and those who will develop relapse. In addition, these exosomes can accurately inform about changes in the patients’ immune status and, in post-therapy follow-up, may alert of impending recurrence. Of various exosome readouts, exosomal PD-L1 reflects early changes discriminating successfully treated from recurrent patients, while TEP and other immunoregulatory features may be useful in post-therapy detection of recurrence. In the future, all identified promising biomarker targets arising from this pilot study will be validated in a big patient cohort.

## Supplementary information

Academic Journals Reporting Checklist

Supplemental material

## Data Availability

All data relevant to the study are included in the article or uploaded as supplementary information.
