# Supplementary material for: Changes in circulating exosome molecular profiles following surgery/(chemo)radiotherapy: early detection of response in head and neck cancer patients
Source: Br J Cancer. 2021 Oct 12;125(12):1677–86. doi: 10.1038/s41416-021-01567-8 (PMC8651659; doi:10.1038/s41416-021-01567-8)
Supplement: Supplementary file 2 — Supplemental material [file 41416_2021_1567_MOESM2_ESM.docx]

**Supplementary data for manuscript “Changes in circulating exosome molecular profiles following surgery/(chemo)radiotherapy: early detection of response in head and neck cancer patients.”**


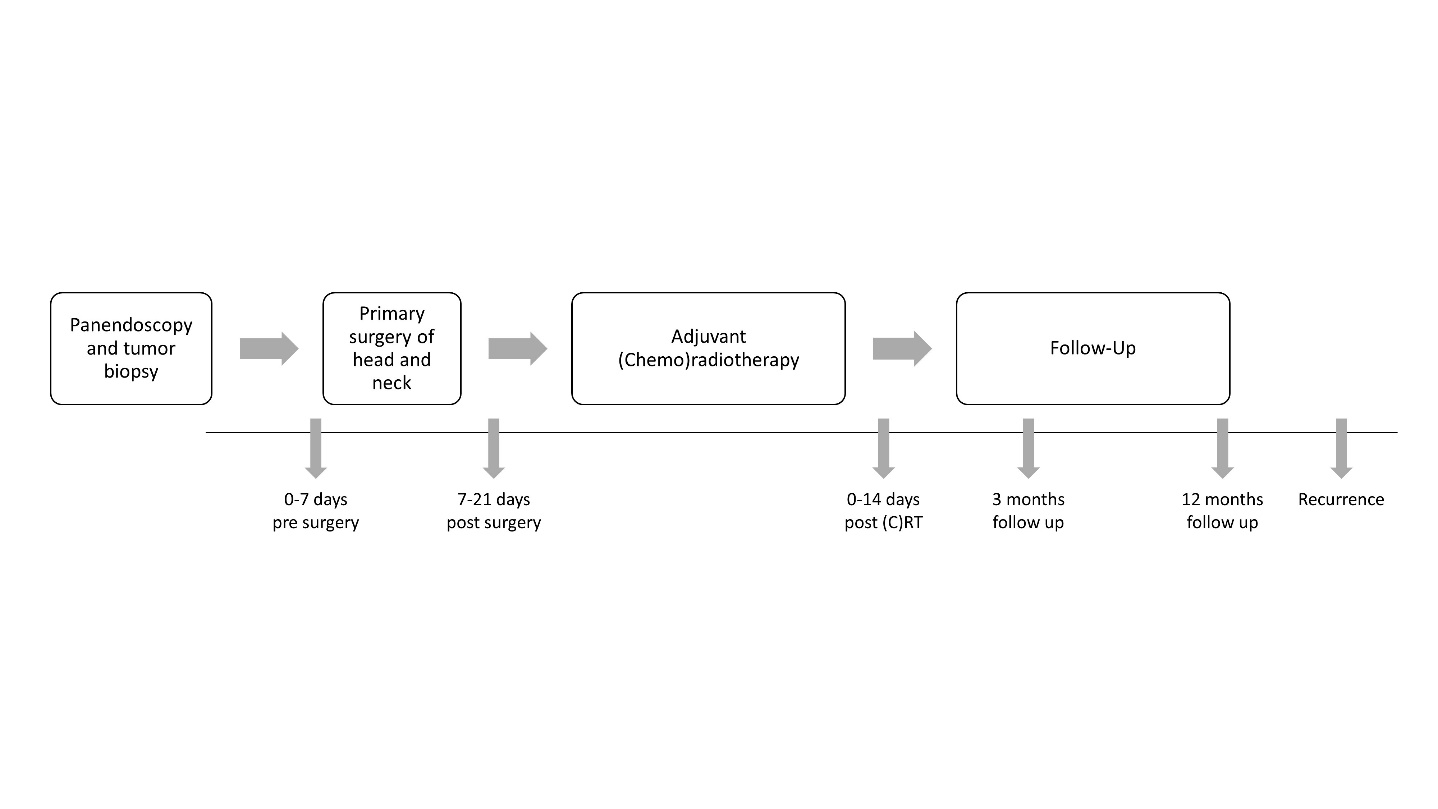


**Supplementary Figure S1: Sampling timeline.**

Blood samples were taken at defined time points before, during, and after curative treatment as well as at time of recurrence. Pre-surgery corresponds to baseline and post-surgery equals pre-(C)RT. (C)RT = (Chemo)radiotherapy


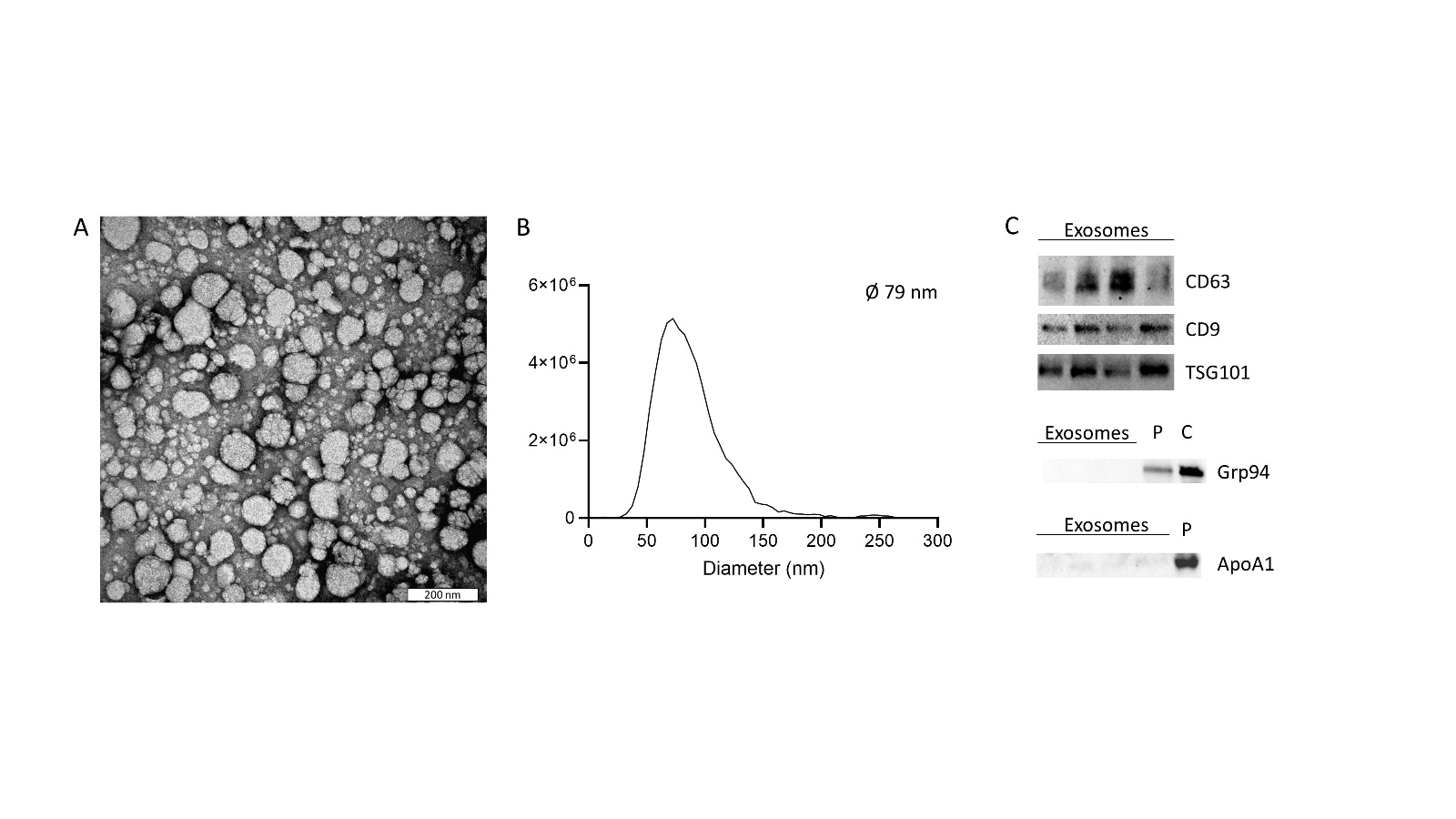


**Supplementary Figure S2: Characterization of exosomes isolated from plasma.**

(A) Representative transmission electron microscopy (TEM) image of exosomes. Scalebar = 200 nm. (B) Representative size distribution of exosomes measured by nanoparticle tracking analysis (NTA). (C) Exosomes isolated from plasma of HNSCC patients were analyzed by Western blot for the presence of exosome specific markers using antibodies against CD63 under non-reducing conditions and antibodies against CD9, TSG101, Grp94 and ApoA1 under reducing conditions. Cell lysate (C) and pure plasma (P) were used as positive controls for Grp94 and ApoA1, respectively.


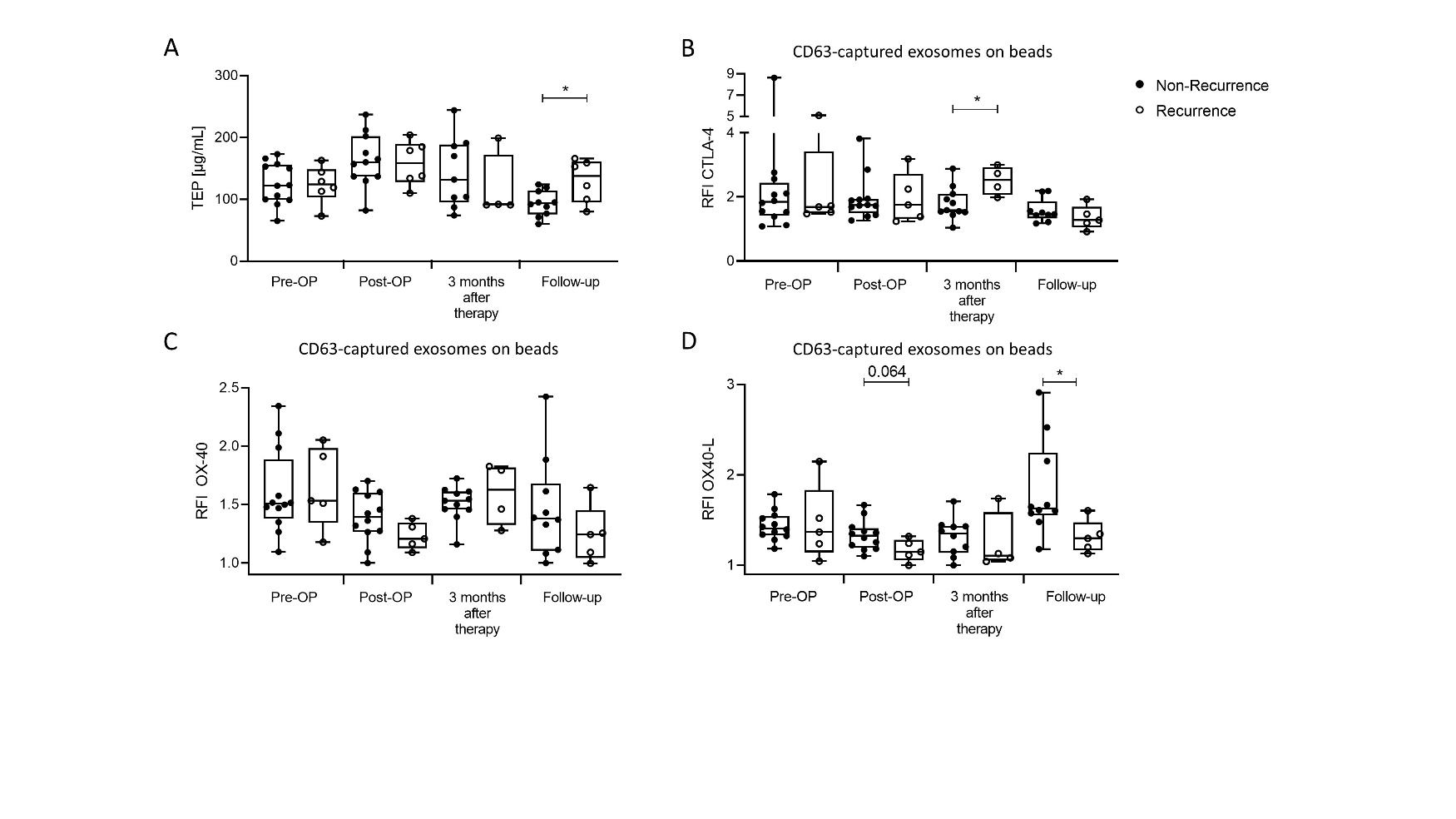


**Supplementary Figure S3: TEP, CTLA-4, OX-40 and OX40-L on exosomes isolated from plasma of non-recurrent and recurrent HNSCC patients.**

(A) TEP concentrations of the exosome fraction obtained from plasma of n = 12 successfully treated and n = 5 recurrent HNSCC patients was measured by BCA assay. (B-D) Exosomes from n = 12 successfully treated and n = 5 recurrent HNSCC patients were captured using biotinylated CD63-antibodies and stained with fluorochrome-conjugated antibodies against CTLA-4, OX-40 and OX40-L. Surface values as determined by on bead-flow cytometry are shown as RFI compared to an appropriate isotype control. Results are plotted as box-and-whisker blots representing the median value, the 25th and 75th quartiles and the range. *P* values were determined by Mann-Whitney test, with * corresponding to *p* ≤ 0.05.
